# Editorial: Artificial Intelligence in Traditional Medicine

**DOI:** 10.3389/fphar.2022.933133

**Published:** 2022-08-04

**Authors:** Chaoyong Wu, Jianxin Chen, Elaine Lai-Han Leung, Hang Chang, Xu Wang

**Affiliations:** ^1^ School of Life Sciences, Beijing University of Chinese Medicine, Beijing, China; ^2^ State Key Laboratory of Quality Research in Chinese Medicines, Faculty of Chinese Medicine, Macau University of Science and Technology, Macao, China; ^3^ Lawrence Berkeley National Laboratory, Berkeley, CA, United States

**Keywords:** ethnopharmacology, traditional medicine, artificial intelligence, machine learning, traditional Chinese medicine, network pharmacology

Traditional medicine (TM) has good efficacy in treating various diseases and has the potential to promote global health. A well-known example, artemisinin has played an important role against malaria and saved millions of lives worldwide (Tu, 2011), and Realgar-*Indigo naturalis* (Chinese medicinal prescription with *Indigo naturalis*, realgar, and *Salvia miltiorrhiza* as the main components, and tetra-arsenic tetra-sulfide, tanshinone IIA, and indirubin as primary active ingredients) has been effective in treating acute promyelocytic leukemia (Wang et al., 2008; Zhu and Huang, 2014). Despite having been practiced for thousands of years with a rich history of practical experience, TM has been insufficiently exploited and remains mysterious to a certain extent, due to complex prescriptions and a lack of objective evaluation standards. While previous TM research has been successful, it is not efficient in new drug development presently and it could take years to investigate the activity of TM, meaning that speeding up TM mechanism research is a significant challenge.

As an emerging discipline, artificial intelligence (AI) technologies have been enthusiastically explored by TM researchers in recent years. AI-powered methods, such as machine learning, deep learning, network pharmacology, bioinformatics, systems biology, chemical informatics, and computer vision, can link chemical composition, herbal medicine, drugs, targets, symptoms, and diseases. In other words, AI provides new approaches to exploring ancient literature on TM, enabling the screening of major components of herb or formula, revealing the mechanism of action, and guiding the precise use of TM. The present Research Topic aims to discover novel approaches and strategies for developing and evaluating medicines *via* AI technologies. Based on initial validation, peer review, and specialty chief editor evaluation, eight manuscripts were accepted from a total of 57 submissions to this “*Artificial Intelligence in Traditional Medicine*” Research Topic.

Wang et al. used the optimized support vector machine algorithm to construct a serological-based lung cancer diagnosis model, analyzed the potential therapeutic mechanism of wogonin [a component of *Scutellaria baicalensis* Georgi (Lamiaceae)] on lung cancer, explored the relationship between serological markers and the target of wogonin, and finally constructed a signaling pathway of wogonin regulation. Combining the diagnosis and treatment of lung cancer using a machine learning algorithm, network pharmacological analysis, and *in vitro* experiments, this study may provide a new approach to uncovering the diagnostic basis and therapeutic mechanisms of TM in lung cancer.

Acute respiratory distress syndrome (ARDS) is a disease with high mortality and lacks effective pharmacological therapy. Integrating network pharmacology, AI, and lung tissue transcriptome analyses, Lu et al. found that Ning Fei Ping Xue (NFPX) decoction could treat ARDS and modulate immune-inflammation response by regulating the gene expression level of HRAS, AMPK, and SMAD4. NFPX decoction is comprised of 20 herbs (e.g., *Paeonia lactiflora* Pall. (Paeoniaceae) and *Atractylodes macrocephala* Koidz. (Asteraceae)] with 37 active ingredients (e.g., astragaloside IV and neochlorogenic acid). Their findings highlight the efficacy and mechanism of NFPX decoction on ARDS.

*Penthorum chinense* Pursh (Penthoraceae) (*P. chinense*) is a traditional Chinese medicine (TCM) for treating liver injuries. Du et al. developed an efficient LC-MS method, combing data-independent acquisition and data-dependent acquisition to fully investigate the components of *P. chinense*. Network pharmacology analysis was then applied to reveal the active mechanism of *P. chinense*, which was also verified by *in vivo* experiment. In summary, this scheme provides a framework for studying TCM chemical components and active mechanisms.

Cai et al. provided an important scheme for investigating the therapeutic ingredients and mechanisms of *Isatis tinctoria L.* (Brassicaceae] (Banlangen) against the Influenza A virus. The network-based framework successfully identified six active anti-influenza candidates (acacetin, eupatorin, dinatin, indirubin, linarin, and tryptanthrin) after validation by *in vitro* experiments. This scheme of in silico prediction combined with experimental validation could serve as a valuable and complementary approach to accelerate the development of novel anti-Influenza A virus agents.

He Xue Ming Mu tablet (HXMM) is a TCM approved by the China Food and Drug Administration for treating retinal degenerative diseases. HXMM consists of 19 herbs [e.g., Typha domingensis Pers. (Typhaceae) and *Salvia miltiorrhiza* Bunge (Lamiaceae)] with 13 active components (e.g., germacrone and nicotinic acid). Xi et al. used a quantitative algorithm to evaluate the disturbance of drugs on the disease network and explore the specific mechanism of multi-target HXMM. HXMM can reduce network robustness on inflammatory and oxidative stress subnetworks to exhibit anti-inflammation/oxidation activity. A combination of bioinformatics prediction and experiment validation provides a framework for the precision application of TCM.

There are two review papers in the Research Topic. Wang et al. systematically review the application of deep learning and cloud computing in medicine and discuss the potential application of AI in the diagnosis, active drug screening, elucidation of the pharmacological mechanism of TCM, prognosis, and efficacy assessment of rheumatoid arthritis. A systematic scoping review by Chu et al. revealed the use of AI in various complementary and alternative medicine categories, such as herbal medicine, tongue and lip diagnoses, and acupuncture intervention. The authors believed that AI techniques could assist medical personnel to better serve the patients. These two review papers may provide technological knowledge for efficiently developing TM-related treatments.

Shi et al. developed a new method of fatigue classification by integrating pulse data and tongue images with machine learning algorithms. Specifically, four machine learning models, including a support vector machine, logistic regression, random forest, and neural network, were used to conduct classification experiments for disease fatigue versus non-disease fatigue classification. This work may provide opportunities for future comprehensive assessments of individual health status and non-invasive ethnopharmacological evaluation.

To summarize, the papers in this Research Topic showcase the application of AI in TM, helping readers understand the usefulness of AI-powered methods and encouraging them to design effective models in pharmacology research for extracting more information from open databases and private experimental results.

## References

[B1] Tu Y. (2011). The discovery of artemisinin (qinghaosu) and gifts from Chinese medicine. Nat. Med. 17 (10), 1217–1220. 10.1038/nm.2471 21989013

[B2] Wang L. Zhou G. B. Liu P. Song J. H. Liang Y. Yan X. J. (2008). Dissection of mechanisms of chinese medicinal formula realgar-indigo naturalis as an effective treatment for promyelocytic leukemia. Proc. Natl. Acad. Sci. U. S. A. 105 (12), 4826–4831. 10.1073/pnas.0712365105 18344322 PMC2290784

[B3] Zhu H. H. Huang X. J. (2014). Oral arsenic and retinoic acid for non-high-risk acute promyelocytic leukemia. N. Engl. J. Med. 371 (23), 2239–2241. 10.1056/NEJMc1412035 25470713

